# Magnitude of health expenditure induced removable poverty in India: Some reflections of Ayushman Bharat

**DOI:** 10.1016/j.heliyon.2023.e23464

**Published:** 2023-12-13

**Authors:** Ramna Thakur, Mohammad Ahmad Faizan

**Affiliations:** School of Humanities and Social Sciences, Indian Institute of Technology Mandi, 175005, India

**Keywords:** Household, Removable, Poverty, Health, Expenditure, Healthcare

## Abstract

The authors have measured the health expenditure-induced removable poverty in India using nationally representative consumer expenditure surveys of three quinquennial rounds conducted by the National Sample Survey Organization (NSSO). This study has also focused on the reflections of Ayushman Bharat-Pradhan Mantri Jan Arogya Yojana (AB-PMJAY), the world's largest Government-funded health insurance scheme, on these poverty rates in the country. The study has used headcount, payment gap, and concentration index to measure the economic burden and impoverishment impact of out-of-pocket (OOP) health expenditure. The analysis shows that the incidence and depth of poverty are substantially understated because of overlooking OOP health expenditure in the country's standard poverty measure. Outpatient care contributes almost four times more than inpatient care to health expenditure-induced impoverishment in India, though this care has not been covered in the AB-PMJAY. Muslims, among all religious groups, Scheduled Castes among social groups, and casual labourers among different household types are more vulnerable to OOP health expenditure-induced removable poverty in the country. Poverty, in general, has dropped significantly, but the share of health expenditure-induced poverty in general poverty has increased substantially. It has risen considerably in rural areas and among India's most vulnerable sections of society in the past 20 years. We emphasised that universal health insurance coverage is needed in India. Implementing comprehensive health insurance schemes that cover both inpatient and outpatient care can help alleviate the financial burden of healthcare expenses on households and contribute to reducing poverty rates.

## 1 I. introduction

Individuals' health has always been considered an indicator of prosperity and happiness [1]. Even today, health is the critical determinant and measuring stick of human well-being worldwide [2,3]. Generally, an individual's health is defined as a state of complete physical, mental, and social well-being and not merely the absence of infirmity [3,4]; Organization, 2006; [5]. To ensure individuals' well-being, equitable access to healthcare services is a fundamental right of human beings and a critical goal for healthcare providers [6].

Indeed, while all governments may agree in principle that providing better healthcare systems is crucial for ensuring equitable access to care for everyone, the actual implementation of such systems is often challenging, particularly in Low- and Middle-Income Countries (LMICs) [7,8]. In reality, the majority of the population in these countries cannot access healthcare services, and even if they could, it is not affordable. Poor health insurance coverage and inadequate contribution of the public healthcare system compel people to spend money by sacrificing other basic needs. There is ample evidence showing that this out-of-pocket (OOP) health expenditure forces people to reduce their household expenditure on different basic needs such as food, clothing, shelter and education of their children. It leads them to significant financial hardship, often pushing them into deep poverty [[9], [10], [11]]. Many times, because of the same reason, people postpone their healthcare needs, which further increases healthcare costs and sometimes reduces the chances of survival from illness, which also deteriorates the financial condition of the households [9].

The impact of health expenditure on impoverishment is a well-researched and extensively discussed topic within and across countries. If health expenditure is completely non-discretionary, then the difference between poverty estimates derived from gross households’ resources and those calculated after deducting health expenditure can be interpreted as poverty due to health expenditure [[12], [13], [14], [15], [16], [17], [18]]. Numerous studies have examined the relationship between health expenditure and poverty, particularly in low- and middle-income countries where the burden of out-of-pocket health costs tends to be more pronounced.

Researchers and policymakers have also tried to explain this relationship in India by conducting different empirical studies. They agreed that out-of-pocket (OOP) health expenditure profoundly affects India's impoverishment, where the government's healthcare spending is 1.28 % of the GDP [13,19]. Health expenditure is one of the primary causes of household debt and poverty in India's low and middle-income groups [16]. More than 70 % of the expenditure on health is out-of-pocket (OOP) of individuals [[20], [21], [22], [23], [24], [25]]. Health insurance would have financially protected the households in this situation, but only 1.6 % of the population is covered under health insurance [26]. Ultimately, out-of-pocket spending becomes the crucial option that pulls households into poverty and suffering [27]. There are many studies like [17,18,[28], [29], [30], [31]] [21]; and [32] which revealed that in other low and middle-income countries (LMICs) also, there is a heavy reliance on OOP health expenditure. Its share in total spending constitutes three-fifths in Bangladesh, Malawi, Bhutan and China, three-fourths in Vietnam and Nepal and more than half in Ghana and Kenya.

In India, several studies have analysed the impoverishment impact of OOP health expenditure. Studies like [25,28,[33], [34], [35]]; and [36] have shown that the frequency of households falling below the poverty line due to OOP health expenditure varies between 3 % and 7 % in the country. Some other studies such as [22,34,[37], [38], [39], [40]]; and [41] have found that the incidence of poverty due to OOP health expenditure is higher in rural areas and poor states compared to urban areas and rich states in India. Few more studies like [16,18,33,42,43]; and [21] have found that apart from rural-urban differentials, the financial burden associated with OOP health expenditure and resultant impoverishment has increased among the disadvantaged sections of the society as compared to advantaged ones. Studies like [[44], [45], [46]] have also reported the effects of different socioeconomic covariates such as religion, social category and household type on OOP health expenditure and its impoverishment impact in India.

The studies above on the impoverishment impact of OOP health expenditure have supported the fact that critical health expenditure pushes households into poverty. These studies comprehensively indicated the scale of impoverishment due to health expenditure and have shown the extent to which poverty is underestimated or hidden. These studies focused on the population above the poverty line because of more healthcare spending. This hidden poor population can be brought out of poverty if there is universal health coverage (UHC), as we believe that the amount they must spend on health care will be spent on other minimum food and non-food consumption. There is ample evidence which shows that for financing healthcare, households sometimes discontinue their children's education, reduce the level of food consumption and shift out families from better rental apartments to cheaper ones [14,[47], [48], [49], [50]]. These strategies induce long-term adverse outcomes regarding future efficiency and productivity reduction.

However, besides the above-mentioned health expenditure-induced ‘Hidden Poverty’ (HP), another dimension called ‘Reinforced Poverty’ (RP) also needs attention. It examines the number of poor individuals who go into deep poverty because of OOP health expenditure. As this population is already below the poverty line, any spending on health care forces them to compromise on their other critical food and non-food consumption needs. This percentage of people is below the poverty line, but no cause is defined. In their study [41], have considered this dimension of poverty.

Furthermore, in this reinforced poverty, a certain percentage of the population is below the poverty line by the same or less amount they spend on their critical health care. This population percentage can also be brought out of poverty, like hidden poverty, if there is UHC in the country. We call this poverty ‘Removable Reinforced Poverty’ (RRP). The UHC will allow them to spend on consumption the amount they currently spend on critical healthcare. It will increase their minimum consumption expenditure and bring them above poverty. Calculating the population going into deep poverty because of OOP health expenditure and its share, which can be carried out of poverty, is as important as the calculation of ‘hidden poverty. Both types of poverty together constitute the total poverty impact of health expenditure.

It is clear from the literature that general poverty has been declining rapidly over the years, while ill-health expenditure-induced poverty as a share of general poverty has shown an increasing tendency. Health expenditure hides people's poverty above the poverty line and causes the deepening of poverty for a substantial percentage of the population below the poverty line. The existing literature has focused mainly on hidden poverty, except for a study by Ref. [41]; which analysed reinforced poverty. Still, there is a lack of studies focused on removable reinforced poverty, a critical dimension to gauge the total poverty impact of OOP health expenditure. Considering these issues, this study aims to estimate the magnitude of health expenditure-induced poverty, which can be removed if there is UHC in India. This study has two objectives: 1) to examine the magnitude of health expenditure-induced removable poverty in India and 2) to study the role of AB-PMJAY in reducing the share of the population which goes into poverty because of OOP health expenditure.

This paper contributes to the literature in two important ways. First, it explains how researchers can calculate the total health expenditure-induced removable poverty figures. These poverty figures are significant for any policy intervention for a particular region or country. Focusing on the population which is above the poverty line just because of high OOP health expenditure (we call it ‘hidden poverty’) only gives underestimated figures of ill health expenditure-induced poverty. To gauge the share of the total population whose poverty status can be changed with an intervention like UHC, it becomes essential to calculate the total ‘removable poverty’ figures. It is the proportion of the population which can be brought out of poverty (in the conventional method of poverty measurement). Second, this paper has also focused on the implication of AB-PMJAY, the world's largest government-funded health assurance scheme, on these poverty rates in the country. This study has suggested how this scheme can be more effective in reducing the share of the population that goes into poverty due to OOP health expenditure.

The study's outline is as follows: the methods described in the next section (section II). Section III presents the empirical results of ill-health-induced removable poverty. Section IV includes a discussion, section V presents the conclusion, and section VI consists of the study's limitations.

## 2 Methodology

This study revolves around two categories of individuals in the country. The first includes those above the poverty line merely because of high spending on healthcare, which pulls them above the poverty line. This category of individuals is called hidden poor as they are above the poverty line in the general measurement of poverty. It is imperative to mention that health expenditure is one component of total consumption expenditure, which is India's poverty measurement base.

Second is the category of individuals below the poverty line by the same or less amount they spend on their critical health care pushes them into deep poverty. This category of individuals is below the poverty line, but no reason is defined. This category of individuals is called removable reinforced poor in this study. People in hidden and removable reinforced poor are different, and their addition does not lead to double counting.

## 3 II.1 data

This study is based on three quinquennial consumer expenditure surveys (CES) conducted by the National Sample Survey Organization (NSSO). The number of households surveyed during these rounds is 120,309 households consisting of 71,385 rural and 48,924 urban in 1999–2000, 124,644 consisting of 79,298 rural and 45,346 urban in 2004–05, 101,662 consisting of 59,695 rural and 41,967 urban households in 2011-12 respectively. The surveys adopted a stratified multistage sample design, using census villages for the rural areas and urban blocks for the urban areas as the first-stage units and households as the second-stage units. We have used the household as the unit of study and adjusted all estimates according to their respective weights.

This study has analysed the magnitude and dimensions of health expenditure-induced removable poverty in rural, urban, different social groups [Scheduled Castes (SCs), Scheduled Tribes (STs), Other Backward Classes (OBCs) and General], household types [self-employed, salaried, casual labour and others] and religious groups [Hindu, Muslims, and others] in India after applying the respective weights. ‘Mixed reference period’ has been used in all three rounds in this study.

In the ‘Mixed Reference Period’, the last 365 days of data for commodities like clothing, bedding, footwear, education, medical (institutional), and durable goods and the last 30 days for the rest of the items have been used [51]. To minimise recall errors, a very detailed item classification has been adopted by NSSO to collect information, including 142 items of food, 15 items of energy (fuel, light and household appliances), 28 items of clothing, bedding and footwear, 19 items of educational and medical expenses (non-institutional), 51 items of durable goods, and 89 other items [52].

The survey provided information for inpatient (institutional) care (last 365 days recall period) and outpatient (non-institutional) care (last 30 days recall period) separately in health expenditure. The distinction between inpatient and outpatient medical expenditure lies in whether the expenditure was incurred on medical treatment as an inpatient of a medical institution or otherwise. The medical institution here covers private and government institutions such as hospitals and nursing homes. Expenditure on inpatient care was converted into monthly figures to calculate out-of-pocket expenditure on health care.

Medical expenditure in the case of inpatients includes the purchase of drugs and medicines, expenditure incurred on clinical tests such as pathological tests, ECG, X-ray, etc.,^1^ professional fees of doctors, nurses, etc.,^1^ payments made to hospitals and nursing homes for medical treatment and ‘other health expenditures’ which were not recorded above. Outpatient spending includes expenditure on all family planning devices, including medical termination of pregnancy, along with the same categories mentioned in the inpatient category except for hospital/nursing home charges.

It is essential to mention here that the NSS-CES surveys neither include non-prescription drugs in the medical expenditure category nor include spending on medical equipment, such as glasses, contact lenses, hearing aids and orthopaedic equipment (these are recorded as durables), including dental costs [52]. That is why we call it critical health expenditure, which is highly underestimated in the country.

In this study, the official poverty line suggested by the Planning Commission, Government of India [53] has been used as a yardstick to measure poverty headcount after adjusting to the Consumer Price Index (CPI) for 1999–2000, 2004-05 and 2011–12. As per this poverty line, INR 1000 for urban and 816 for rural areas for 2011-12 per capita per month is the minimum consumption expenditure (on the above items), which must be non-poor. Households with consumption expenditures below the poverty line are considered poor, while the rest are non-poor. Using a purchasing power parity (PPP), the exchange rate of US $1 = 15.11 rupees (see World Bank, 2014) corresponds to US $54 per month in rural areas and US $66.18 in urban areas of the country. We have adjusted these figures to the other years using the respective year's price index.

## 4 II.2 methods

The study starts by examining hidden poverty, then moves on to analysing removable reinforced poverty, and finally employs logistic regression to determine the likelihood of an individual falling into poverty due to out-of-pocket (OOP) health expenditures.

### 4.1 Measurement of the poverty impact of OOP health expenditure

This study calculates OOP health expenditure-induced removable poverty in health expenditure-induced hidden and reinforced poverty**.** To measure health expenditure-induced hidden poverty, the methodology suggested by Ref. [18] has been adopted, while removable reinforced poverty has been calculated in the given ways.

### 4.2 Hidden poverty

The difference between the relevant poverty measures before and after paying for health care is the poverty impact of OOP payments for health. These are given as follows:

(1) $$PI_{H}=P_{H}^{post}-P_{H}^{pre}$$

where $PI_{H}$ can be called healthcare expenditure-induced hidden poverty.

The above equation represents the difference between the poverty headcount before and after paying for health care. It means the population's proportion is above the poverty line or non-poor only because of high OOP expenditure on critical health care, which pushes their total expenditure above the poverty line. Otherwise, they are poor.

To calculate health care induced hidden poverty, we estimated individual i's pre-payment and post-payment total consumption, which we denoted with x_i_ and compared with $z_{pov}^{pre}$ and $z_{pov}^{post}$ which are pre-payment and post-payment poverty lines.

$${P_{h}}_{i}^{pre}=1,\text{if}\;\text{xi}<z_{pov}^{pre}$$

Further, it is essential to clarify that in this study, we have used a pre-payment poverty line $z_{pov}^{pre}$ and the post-payment poverty line $z_{pov}^{post}$ and all superscripts ‘pre’ by the superscript ‘post’ to give the analogous to the post-payment measures (Wagstaff, 2003).

In this way, the pre-payment headcount is equal to

(2) $$P_{H}^{pre}=\frac{1}{N}\sum_{i=1}^{n}P_{h_{i}}^{pre}=\mu_{Hp}^{pre}$$

Where N is the sample size and ${P_{h}}_{i}$ is the poverty headcount. In the same way, it is calculated for the post-payment.

To measure the hidden poverty gap or the average amount by which consumption expenditure falls short of the poverty line, we have used the following equation:

(3) $$GI_{H}=G_{H}^{post}-G_{H}^{pre}$$

This equation represents the difference between poverty gaps before and after health payments, which is the hidden poverty gap. This gap has been calculated as follows:

(4) $$G_{H}^{pre}=\frac{1}{N}\sum_{i=1}^{n}{{G_{h}}_{i}}^{pre}=\mu_{Hg}^{pre}$$

where ${G_{h}}_{i}^{pre}$ is the pre-payment hidden poverty gap, which is equal to $x_{i}-z_{pov}^{pre}$ if $x_{i}<z_{pov}^{pre}$ and zero otherwise. In the same way, it is calculated for the post-payment hidden poverty gap.

With the following equation's help, we calculated the mean positive gap (MPGI_H_) of hidden poverty, which shows the average poverty gap of only those individuals (hidden poor) who are poor but above the poverty line because of high OOP health expenditure.

(5) $$MP{GI}_{H}=MPG_{H}^{pre}-MPG_{H}^{post}$$

(6) $$MPG_{H}^{pre}=\frac{\sum_{i-1}^{n}{G_{h}}_{i}^{pre}}{\sum_{i=1}^{n}{{P_{h}}_{i}}^{pre}}=\frac{\mu_{Hg}^{pre}}{\mu_{Hp}^{pre}}$$

In the same way, it is also calculated for post-$MPG_{H}^{post}$.

After calculating MPGI_H_, we have also calculated the normalised poverty gap (NPGI_H_) of hidden poverty, which helps in international comparison. We have figured it out with the help of the following formula:

(7) $$NP{GI}_{H}=NG_{H}^{post}-NG_{H}^{pre}$$

This equation shows the difference between the normalised poverty gap before and after OOP health payments, which is normalised poverty. It is calculated as follows.

(8) $$NG_{H}^{pre}=\frac{G_{H}^{pre}}{z_{pov}^{pre}}$$

In the same way, it is calculated for post-payment also.

Ill health expenditure-induced removable reinforced poverty is the number of individuals below the poverty line and going into deep poverty because of OOP health expenditure. Like hidden poor in the study, this share of the population can also be brought out of poverty (if measured with the conventional method of poverty, e.g. per capita per month minimum given consumption expenditure) if there is UHC in the country, has been calculated as follows.

### 4.3 Removable reinforced poverty

We know from the above equations that for an individual:

${G_{h}}_{i}^{pre}=x_{i}-z_{pov}^{pre}$, if $x_{i}<z_{pov}^{pre}$ and zero otherwise. Hence, we have calculated the remaining poverty gap, ${G_{r}}_{i}^{post}=x_{i}-z_{pov}^{pre}+h_{i}$ , if $x_{i}<z_{pov}^{pre}$ and $x_{i}-z_{pov}^{pre}+h_{i}<0\;$ and zero otherwise. For headcount, $P_{r_{i}}^{post}$ = 1 if $x_{i}-z_{pov}^{pre}+h_{i}$ <0, and zero otherwise. $P_{r_{i}}^{post}$ = remaining poverty level of the ith individual after topping up health expenditure, and we call it ‘remaining reinforced poverty’, h_i_ = health expenditure of the ith individual. We want to make clear here that the poverty headcount of remaining reinforced poor is the total of individuals who are below the poverty line with ${G_{r}}_{i}^{post}$ < 0, and removable reinforced poverty with ${G_{r}}_{i}^{post}$ > 0.

For the sample, it is calculated as follows:

(9) $$P_{R_{r}}^{post}=\frac{1}{N}\sum_{i=1}^{n}P_{r_{i}}^{post}=\mu_{Rp}^{post}$$

Where; $\sum_{i=1}^{n}P_{r_{i}}^{post}$ = total number of remaining reinforced poor after deducting health expenditure,

In this case, we have calculated the remaining reinforced poverty gap, which is equal to $x_{i}-z_{pov}^{pre}+h_{i}$ if $x_{i}<z_{pov}^{pre}$ and $x_{i}-z_{pov}^{pre}+h_{i}$ <0. For the sample, it is calculated as follows:

(10) $$G_{R_{r}}^{post}=\frac{1}{N}\sum_{i=1}^{n}G_{r_{i}}^{post}=\mu_{Rg}^{post}$$

After calculating the remaining reinforced gap, we have calculated the removable reinforced gap:

(11) $$GI_{R}=G_{H}^{pre}-G_{R_{r}}^{post}$$

We have also calculated the normalised removable reinforced poverty gap as follows:

(12) $$NP{GI}_{R}=NG_{H}^{pre}-NG_{R_{r}}^{post}$$

This equation shows the difference between normalised removable reinforced poverty gaps before and after deducting OOP health expenditure. It is calculated as follows:

(13) $$NG_{R_{r}}^{post}=\frac{G_{R_{r}}^{post}}{z_{pov}^{post}}$$

We have also calculated the removable reinforced mean poverty gap as follows:

(14) $$MP{GI}_{R}=MPG_{H}^{pre}-MPG_{R_{r}}^{post}$$

Which is calculated as follows:

(15) $$MPG_{R_{r}}^{post}=\frac{\sum_{i=1}^{n}G_{r_{i}}^{post}}{\sum_{i=1}^{n}P_{r_{i}}^{post}}or\frac{\mu_{Rg}^{post}}{\mu_{Rp}^{post}}$$

If N is the total number of persons in the sample, an estimation of ‘removable reinforced poverty’ of OOP health expenditure is calculated as follows:

(16) $$PI_{R}=\frac{1}{N}\sum_{i=1}^{n}{P_{h}}_{i}^{pre}-\frac{1}{N}\sum_{i=1}^{n}P_{r_{i}}^{post}\;\text{or}\;\mu_{Hp}^{pre}-\mu_{Rp}^{post}$$

Where; $PI_{R}$ = removable reinforced poverty, ${{P_{h}}_{i}}^{pre}$ = pre-payment poverty level and $P_{r_{i}}^{post}$ = remaining reinforced poverty.

After calculating removable reinforced and hidden poverty, total ill health-induced removable poverty is calculated as follows:

Total removable poverty of OOP health expenditure:

(17) $$\left(TRP_{HR}\right)=PI_{H}+PI_{R}\;\text{or}\;\left(\mu_{Hp}^{post}-\mu_{Hp}^{pre}\right)+\left(\mu_{Hp}^{pre}-\mu_{Rp}^{post}\right)$$

### 4.4 Multivariable logistic regression

The association between socioeconomic covariates and probabilities of incurring impoverishment impact OOP health expenditure has been modelled using multivariable logistic regressions. In our model, the dependent variable is a binary variable that shows an individual's likelihood of going into poverty due to OOP health expenditures.

The mathematical form of multivariate logistic regression has been written as

$$\left(\frac{P}{1-P}\right)=B_{0\;}+B_{1}X_{1}+B_{2}X_{2}+B_{3}X_{3}+u_{i}\text{,}$$

Where P is the probability of health expenditure-induced impoverishment, 1-P is the probability of no health expenditure-induced impoverishment. X_1_ = Religion, X_2_ = Social group, X3 = Household type and u_i_ is the random disturbance term, B_0_ to B_3_ are the estimated parameters. A more meaningful interpretation of the results has been made through the odds ratio. The odds ratio has been obtained by taking the antilog of various slope coefficients as, $\left(\frac{p}{1-P}\right)={\frac{1+e^{Z}}{1+e^{-Z}}=e}^{Z},$ where $\left(\frac{p}{1-P}\right)$ = is the odds ratio of going into poverty due to health expenditures, Z = B_0_ + B_1_X_1_ … … … … …. + B_3_X_3_, e^Z^ is the antilog of Z. Then, by taking the natural log of the above equation, we obtained the logit function, written as $L=In\;\left(\frac{P}{1-P}\right)=Z=B_{0\;}+B_{1}X_{1}+----+B_{3}X_{3}\text{,}$ where L is the log of the odds ratio.

### 4.5 Covariates of logistic regression

The impoverishment impact of OOP health expenditure depends on various socioeconomic covariates. In this study, the dependent variable is a binary variable that shows whether a household is OOP health expenditure induced poor. While religion, social category and household type are the independent variables. Religion^2^ has been divided into Hinduism, Islam, and other religions (Christianity, Sikhism, Buddhism, Zoroastrianism and others). Social categories^3^ have been divided into Scheduled Tribes (STs), Scheduled Castes (SCs), Other Backward Classes (OBCs) and general. The household type has been defined based on the sources of earning a livelihood, including regular/salaried, casual/agricultural, self-employed, and others.

In this study, we have calculated: a) Poverty headcount, which is the proportion of the population going into poverty (removable hidden and reinforced) because of critical OOP health expenditure along with the contribution of inpatient and outpatient care in the removable poverty headcount. b) The Poverty gap is the average amount by appalling which resources fall short of the poverty line. Contribution of inpatient and outpatient care in the removable poverty gap. c) Mean poverty gap, the average shortfall of the affected population only by which resources fall short of the poverty line, along with the contribution of inpatient and outpatient. d) A normalised poverty gap is obtained by dividing the poverty gap by the poverty line, which is very important for international comparison. Besides, we did multivariate logistic regression to know about the social, religious, and economic categories affected most by the burden of OOP health expenditure.

Table 1 provides a detailed description of the study sample's demographic characteristics and health expenditure in all three waves of data. Including variables like sector, religion, social group, and household type in this table allows researchers to comprehensively overview the participants' backgrounds and understand the study population's composition. Table 2 explains the share of different components in the total health expenditure at rural, urban and all India levels, separately for inpatient and outpatient care. Table 3, Table 4 detail health expenditure-induced removable poverty headcount and gap. Table 5 explains the logistic regression results of health expenditure-induced removable poverty, which shows the probability of individuals going into poverty due to OOP health expenditures.

**Table 1:** Description of the analytic sample in rural, urban and all India, 1999–00, 2004-05 and 2011-12.

Particulars	No. of fsu's (village/blocks) surveyed	Sample no. of households	Sample persons	Estimated no. of persons (00)	Average household size	Percentage of households reporting OOP*	OOP health expenditure as a share of TCE*	Share of inpatient care in OOP health expenditure	Share of outpatient care in OOP health expenditure
1999–00									
Sectors									
Rural	6046	71385	374856	691,767,182	5.03 (5.01–5.06)	70.07 (69.73–70.40)	5.0	22.5	77.5
Urban	4116	48924	225160	232,393,085	4.53 (4.49–4.56)	69.61 (69.20–70.02)	4.4	28.5	71.5
Religion									
Hindus	–	93514	456579	757,166,309	4.81 (4.79–4.84)	69.38 (69.09–69.68)	5.1	24.3	75.7
Muslims	–	14607	83157	115,496,152	5.62 (5.53–5.71)	72.83 (72.11–73.55)	5.2	21.4	78.6
Others	–	12071	59721	50,608,663	4.73 (4.66–4.81)	70.20 (69.39–71.02)	4.7	31.1	68.9
Caste									
General	–	49634	245300	337,404,193	4.89 (4.84–4.93)	72.21 (71.81–72.60)	4.9	27.1	72.9
STs	–	13326	66896	80,464,022	4.81 (4.75–4.87)	61.11 (60.28–61.93)	3.9	21.5	78.5
SCs	–	18740	91448	174,641,800	4.81 (4.77–4.86)	70.94 (70.29–71.59)	5.5	20.4	79.6
OBCs	–	38389	195303	330,165,429	4.97 (4.94–5.00)	69.40 (68.94–69.86)	5.4	23.3	76.7
HH Type									
Self employed	–	55145	309477	447,309,389	5.25 (5.18–5.32)	71.84 (71.49–72.19)	5.2	24.5	75.5
Salaried	–	39057	88805	92,631,042	4.68 (4.64–4.73)	68.87 68.23–69.50)	4.2	28.1	71.9
Casual labour	–	11414	141822	299,121,167	4.86 (4.79–4.93)	66.93 (66.34–67.51)	5.5	20.8	79.2
Others	–	14390	58386	83,039,553	4.08 (3.99–4.19)	66.76 (65.86–67.67)	5.6	26.1	73.9
Total	10162	120309	600016	924,160,267	4.90 (4.88–4.92)	69.90 (69.75–69.96)	4.8	24.5	75.5
2004–05									
Sector									
Rural	7999	79298	403207	7,331,055	4.88 (4.86–4.91)	64.97 (64.63–65.30)	6.1	25.9	74.1
Urban	4602	45346	206529	2,485,051	4.36 (4.32–4.40)	64.53 (64.09–64.97)	5.0	27.7	72.3
Total	12601	124644	609736	9,816,106	4.73 (4.72–4.76)	64.41 (64.12–64.86)	5.6	26.5	73.5
Religion									
Hindus	–	95250	455817	8,064,491	4.67 (4.65–4.69)	63.76 (63.46–64.07)	5.6	25.9	74.1
Muslims	–	14791	82156	1,242,225	5.34 (5.27–5.40)	69.70 (68.96–70.45)	5.9	26.5	73.5
Others	–	14603	71712	508666	4.57 (4.50–4.64)	66.65 (65.89–67.42)	5.9	32.3	67.7
Caste									
General	–	41892	201346	3,023,255	4.65 (4.62–4.69)	67.90 (67.45–68.35)	5.5	29.2	70.8
STs	–	16410	81736	847427	4.67 (4.60–4.73)	55.80 (55.04–56.56)	3.8	22.2	77.8
SCs	–	20065	96970	1,922,583	4.71 (4.67–4.76)	65.86 (65.20–66.51)	5.7	21.5	78.5
OBCs	–	46236	229452	4,018,825	4.83 (4.80–4.87)	64.74 (64.30–65.17)	6.1	26.1	73.9
HH Type									
Self employed	–	63500	342323	5,167,431	5.26 (5.23–5.29)	67.33 (66.97–67.70)	5.7	26.5	73.5
Salaried	–	17454	75278	979498	4.21 (4.15–4.27)	63.99 (63.28–64.70)	4.7	28.3	71.7
Casual labour	–	25910	120327	2,877,294	4.52 (4.48–4.56)	60.00 (59.28–60.74)	5.9	22.1	77.9
Others	–	17652	71125	779817	4.00 (3.95–4.07)	62.42 (61.84–63.01)	6.2	28.5	71.5
2011–12									
Sector									
Rural	7469	59695	285796	7,921,166	4.60 (4.57–4.63)	80.81 (80.29–80.92)	7.4	32.0	68.0
Urban	5268	41967	179164	3,168,543	4.05 (4.00–4.09)	78.68 (78.29–79.07)	6.1	34.3	65.7
Religion									
Hindus	–	77062	342138	9,037,259	4.34 (4.31–4.37)	79.50 (79.21–79.78)	6.8	33.1	66.9
Muslims	–	13136	68860	1,512,848	5.11 (5.04–5.19)	84.01 (83.39–84.64)	6.9	30.1	69.9
Others	–	11463	53948	539588	4.30 (4.22–4.37)	77.11 (76.34–77.88)	7.3	35.2	64.8
Caste									
General	–	32408	144579	3,101,366	4.28 (4.22–4.33)	82.37 (81.96–82.79)	6.8	37.2	62.8
STs	–	13629	65103	990428	4.43 (4.35–4.52)	72.27 (71.52–73.02)	4.4	24.0	76.0
SCs	–	15697	71245	2,110,861	4.43 (4.38–4.49)	81.35 (80.74–81.96)	7.1	29.1	70.9
OBCs	–	39914	183973	4,885,893	4.53 (4.49–4.57)	79.69 (79.30–80.09)	7.0	31.3	68.7
HH Type									
Self employed	–	47730	239773	5,654,030	4.97 (4.93–5.00)	81.94 (81.59–82.28)	6.9	33.6	66.4
Salaried	–	27072	117183	1,933,504	4.04 (3.99–4.10)	77.83 (77.33–78.32)	5.8	34.8	65.2
Casual labour	–	19032	85536	3,038,042	4.39 (4.34–4.43)	78.77 (78.19–79.35)	7.2	29.4	70.6
Others	–	7801	22333	461440	2.37 (2.30–2.45)	76.17 (75.22–77.12)	9.1	31.7	68.3
Total	12737	101662	464960	11,089,709	4.43 (4.40–4.46)	79.81 (79.56–80.06)	6.8	32.9	67.1

Note: The vertical total of different socioeconomic categories differs from the total in all waves of data because of the missing values in each category.

**Table 2:** Share of different components in the total health expenditure in rural, urban and all India, 1999–00, 2004-05 and 2011-12.

	Share of inpatient care in total health expenditure					Share of outpatient care in total health expenditure					Share of different components in total health expenditure					
	Medicines	Tests	Fees	Hospital charges	Other medical expenditure	Medicines	Tests	Fees	Family planning	Other medical expenditure	Medicines	Tests	Fees	Hospital charges	Family planning	Other medical expenditure
1999–00																
Rural	12.7	1.5	2.6	3.9	1.8	64.6	2.4	7.5	0.1	2.9	77.2	3.9	10.1	3.9	0.1	4.7
Urban	13.4	2.0	3.8	6.6	2.7	56.3	3.0	9.1	0.2	2.9	69.7	5.1	12.9	6.6	0.2	5.6
Total	12.9	1.7	3.0	4.8	2.1	61.8	2.6	8.0	0.1	2.9	74.7	4.3	11.1	4.8	0.1	5.0
2004–05																
Rural	11.3	2.7	3.8	5.4	2.8	62.7	2.6	7.7	0.1	1.1	74.0	5.3	11.4	5.4	0.1	3.9
Urban	9.9	2.9	4.6	7.3	3.0	56.2	4.5	9.6	0.1	1.8	66.2	7.4	14.2	7.3	0.1	4.8
Total	10.9	2.7	4.0	6.1	2.8	60.4	3.3	8.3	0.1	1.4	71.3	6.0	12.4	6.1	0.1	4.2
2011–12																
Rural	15.0	3.4	3.8	6.2	3.5	53.7	4.1	7.9	0.2	2.2	68.7	7.5	11.7	6.2	0.2	5.7
Urban	12.5	3.7	4.8	9.0	4.3	50.7	4.1	9.2	0.3	1.4	63.1	7.8	14.0	9.0	0.3	5.8
Total	14.0	3.5	4.2	7.3	3.9	52.5	4.1	8.4	0.2	1.9	66.6	7.6	12.5	7.3	0.2	5.8

Note: Figures are based on authors' calculations from NSSO data 1999–2000, 2004-05 and 2011–12.

**Table 3:** Health expenditure induced removable poverty headcount in rural, urban and all India, 1999–00, 2004–05, and 2011-12.

Poverty headcount (in %)	1999–00			2004–05			2011–12		
	Rural	Urban	All India	Rural	Urban	All India	Rural	Urban	All India
Hidden poverty ($PI_{H}=P_{H}^{post}$-$P_{H}^{pre}$)	5.0	3.4	4.6	5.0	3.1	4.5	4.7	2.4	4.1
Hidden poverty as a share of pre-poverty ($PI_{H}$/$P_{H}^{pre}$*100)	10.5	12.9	10.8	11.9	12.2	12.0	18.3	17.5	18.2
Contribution of inpatient care in hidden poverty	1.0	0.7	0.9	0.8	0.7	0.8	1.0	0.7	1.0
Contribution of outpatient care in hidden poverty	4.0	2.7	3.7	4.2	2.4	3.7	3.7	1.7	3.1
Removable reinforced poverty ($PI_{R}=P_{H}^{pre}-P_{Rr}^{post}$)	3.8	2.4	3.5	3.3	1.9	2.9	3.4	1.5	2.8
Removable reinforced poverty as a share of pre-poverty ($PI_{R}$/ $P_{H}^{pre}*100$)	8.0	9.2	8.2	7.9	7.5	7.8	13.1	11.3	12.8
Contribution of inpatient care in removable reinforced poverty	0.6	0.4	0.5	0.3	0.3	0.3	0.5	0.3	0.4
Contribution of outpatient care in removable reinforced poverty	3.2	2.0	3.0	3.0	1.6	2.6	2.9	1.2	2.4
Total removable poverty ($PI_{H}+PI_{R}$)	8.8	5.8	8.1	8.3	5.0	7.4	8.1	3.9	6.9
Total ill-health induced removable poverty as a share of pre-poverty ($PI_{H}+PI_{R}$/ $P_{H}^{pre}*100$)	18.4	22.1	19.0	19.8	19.6	19.9	31.4	28.8	30.9
Contribution of inpatient care in total ill-health-induced removable poverty	18.2	19.5	17.3	13.2	21.0	16.0	19.2	25.8	20.3
Contribution of outpatient care in total ill-health-induced removable poverty	81.8	80.5	82.7	86.8	79.0	83.0	80.8	74.2	79.7

Note: Figures are based on authors' calculations from NSSO data 1999–2000, 2004-05 and 2011–12.

**Table 4:** Health expenditure induced removable poverty gap in rural, urban and all India, 1999–00, 2004-05 and 2011-12.

Poverty gap particulars	1999–00			2004–05			2011–12		
	Rural	Urban	All India	Rural	Urban	All India	Rural	Urban	All India
Poverty Gap (in INR)									
Hidden poverty gap ($GI_{H}=G_{H}^{post}-G_{H}^{pre}$)	7.3	4.7	6.7	6.8	5.6	6.5	10.3	6.1	9.1
Hidden poverty gap as a share of the pre-poverty gap ($GI_{H}$ as a share of $G_{H}^{pre}$)	15.3	16.4	15.5	15.8	15.8	15.8	24.9	22.4	24.3
Contribution of inpatient care in the hidden poverty gap	1.1	1.0	1.2	1.0	0.9	1.0	1.9	1.3	1.8
Contribution of outpatient care in the hidden poverty gap	6.2	3.7	5.5	5.8	4.7	5.5	8.4	4.8	7.3
Removable reinforced poverty gap ($GI_{R}=G_{H}^{pre}-G_{Rr}^{post}$)	4.7	2.9	4.2	4.0	3.3	3.8	5.7	3.6	5.1
Removable reinforced poverty gap as a share of the pre-poverty gap ($GI_{R}$ as a share of $G_{H}^{pre}$)	9.8	10.0	9.8	9.3	9.4	9.3	13.8	13.4	13.7
Contribution of inpatient care in the removable reinforced poverty gap	0.6	0.5	0.6	0.4	0.3	0.4	0.7	0.5	0.7
Contribution of outpatient care in the removable reinforced poverty gap	4.1	2.4	3.6	3.6	3.0	3.4	5.0	3.1	4.4
Total poverty gap ($GI_{H}$ + $GI_{R}$)	11.9	8.3	11.0	10.5	8.6	10.0	16.0	9.7	14.2
Contribution of inpatient care in total poverty gap	14.2	19.7	16.5	13.0	14.4	13.6	16.3	18.6	17.6
Contribution of outpatient care in total poverty gap	85.8	80.3	83.5	87.0	85.6	86.4	83.8	81.4	82.4
Normalised Poverty Gap (in %)									
Normalised hidden poverty gap ($NP{GI}_{H}=NG_{H}^{post}-NG_{H}^{pre}$)	1.8	1.0	1.5	1.5	1.0	1.3	1.3	0.6	0.9
Normalised hidden poverty gap as a share of normalised pre-poverty gap ($NP{GI}_{H}$ as a share of $NG_{H}^{pre}$)	15.3	16.4	15.5	15.8	15.8	15.8	24.9	22.4	24.3
Contribution of inpatient care in the normalised hidden poverty gap	0.3	0.2	0.3	0.2	0.1	0.1	0.2	0.1	0.2
Contribution of outpatient care in the normalised hidden poverty gap	1.5	0.8	1.2	1.3	0.8	1.1	1.1	0.5	0.7
Normalised removable reinforced poverty gap ($NP{GI}_{R}=NG_{H}^{pre}-NG_{R}^{post}$)	1.1	0.6	0.9	0.9	0.6	0.7	0.7	0.4	0.6
Normalised removable reinforced poverty gap as a share of normalised pre-poverty gap ($NP{GI}_{R}$ as a share of $NG_{H}^{pre}$)	9.8	10.0	9.8	9.3	9.4	9.3	13.8	13.4	13.7
Contribution of inpatient care in the removable reinforced poverty gap	0.1	0.1	0.1	0.1	0.1	0.1	0.1	0.1	0.1
Contribution of outpatient care in the removable reinforced poverty gap	1.0	0.5	0.8	0.8	0.5	0.7	0.6	0.3	0.5
Total normalised poverty gap ($NP{GI}_{H}$ + $NP{GI}_{R}$)	2.9	1.6	2.4	2.4	1.6	2.0	2.0	1.0	1.5
Contribution of inpatient care in total normalised poverty gap	13.8	18.8	16.7	12.5	12.5	10.0	15.0	20.0	20.0
Contribution of outpatient care in total normalised poverty gap	86.2	81.3	83.3	87.5	81.3	90.0	85.0	80.0	80.0
Mean Positive Gap (in INR)									
Hidden mean positive gap $MP{GI}_{H}=MG_{H}^{post}$- $MG_{H}^{pre}$)	4.4	3.4	4.3	3.5	4.4	3.6	8.9	8.2	8.7
Hidden mean positive gap as a share of pre-mean positive gap ($MP{GI}_{H}$ as a share of $MG_{H}^{pre}$)	4.4	3.1	4.2	3.4	3.2	3.3	5.5	4.1	5.2
Contribution of inpatient care in the hidden mean positive gap	0.3	0.4	0.5	0.3	0.1	0.3	1.9	1.1	1.6
Contribution of outpatient care in the hidden mean positive gap	4.1	3.0	3.8	3.2	4.3	3.3	7.0	7.1	7.1
Removable reinforced mean positive gap ($MP{GI}_{R}=MG_{R}^{pre}-MG_{Rr}^{post}$)	2.0	0.9	1.8	1.5	2.8	1.7	1.3	4.8	1.8
Removable reinforced mean positive gap as a share of pre-mean positive gap ($MP{GI}_{R}$ as a Share of $MG_{H}^{pre}$)	2.0	0.9	1.8	1.5	2.1	1.6	0.8	2.4	1.1
Contribution of inpatient care in removable reinforced mean positive gap	0.2	0.2	0.2	0.0	0.2	0.1	0.0	0.5	0.2
Contribution of outpatient care in removable reinforced mean positive gap	1.8	0.7	1.6	1.5	2.6	1.6	1.3	4.3	1.6
Total mean positive gap ($MP{GI}_{H}$ + $MP{GI}_{R}$)	6.4	4.3	6.1	5.0	7.2	5.3	10.2	13.0	10.5
Contribution of inpatient care in removable reinforced mean positive gap	7.8	14.0	11.5	6.0	4.2	7.5	18.6	12.3	17.1
Contribution of outpatient care in removable reinforced mean positive gap	92.2	86.0	88.5	94.0	95.8	92.5	81.4	87.7	82.9
Total Gap as a share of Total Consumption Expenditure (in %)									
Hidden poverty gap as a share of total consumption expenditure ($TP{GI}_{H}=$$TG_{H}^{pre}$ - $TG_{H}^{post}$)	1.4	0.5	1.0	1.1	0.5	0.8	0.8	0.3	0.6
$TP{GI}_{H}$ as a share of $TG_{H}^{pre}$ ((in %)	15.3	16.4	15.5	15.8	15.8	15.8	24.9	22.4	24.3
Contribution of inpatient care in hidden poverty gap as a share of total consumption expenditure	0.2	0.1	0.2	0.2	0.1	0.1	0.1	0.1	0.1
Contribution of outpatient care in hidden poverty gap as a share of total consumption expenditure	1.2	0.4	0.8	0.9	0.4	0.7	0.7	0.2	0.5
Removable reinforced poverty gap as a share of total consumption expenditure ($TP{GI}_{R}=$$TG_{R}^{pre}$ - $TG_{R}^{post}$)	0.9	0.3	0.6	0.7	0.3	0.5	0.4	0.2	0.3
$TP{GI}_{R}$ as a Share of $TG_{H}^{pre}$ (in %)	9.8	10.0	9.8	9.3	9.4	9.3	13.8	13.4	13.7
Contribution of inpatient care in removable reinforced poverty gap as a share of total consumption expenditure	0.1	0.1	0.1	0.1	0.0	0.1	0.1	0.1	0.1
Contribution of outpatient care in removable reinforced poverty gap as a share of total consumption expenditure	0.8	0.2	0.5	0.6	0.3	0.4	0.3	0.1	0.2
Total gap as a share of total consumption expenditure ($TP{GI}_{H}$ + $TP{GI}_{R}$)	2.3	0.8	1.6	1.8	0.8	1.3	1.2	0.5	0.9
Contribution of inpatient care in total gap as a share of total consumption expenditure (in %)	13.0	25.0	18.8	16.7	12.5	15.4	16.7	40.0	22.2
Contribution of outpatient care in total gap as a share of total consumption expenditure (in %)	87.0	75.0	81.2	83.3	87.5	84.6	83.3	60.0	77.8

Note: Figures are based on authors' calculations from NSSO data 1999–2000, 2004-05 and 2011–12.

**Table 5:** Logistic regression of health expenditure induced removable poverty (hidden and removable reinforced) in rural, urban and all India, 1999–00, 2004-05 and 2011-12.

Variables	1999–00			2004–05			2011–12		
	Rural	Urban	All India	Rural	Urban	All India	Rural	Urban	All India
Religion									
Hindus	1	1	1	1	1	1	1	1	1
Muslims	1.42* (1.35–1.50)	2.32* (2.18–2.47)	1.68* (1.61–1.75)	1.55* (1.47–1.63)	2.17* (2.04–2.30)	1.83* (1.76–1.91)	1.31* (1.22–1.40)	1.69* (1.57–1.82)	1.50* (1.42–1.57)
Others	0.30* (0.28–0.32)	0.38* (0.34–0.43)	0.31* (0.30–0.33)	0.23* (0.21–0.25)	0.36* (0.33–0.40)	0.26* (0.25–0.28)	0.27* (0.24–0.30)	0.38* (0.33–0.43)	0.30* (0.28–0.32)
Social Group									
General	1	1	1	1	1	1	1	1	1
STs	3.36* (3.18–3.56)	2.36* (2.12–2.63)	3.51* (3.35–3.68)	3.82* (3.61–4.05)	2.26* (2.04–2.51)	3.38* (3.22–3.55)	4.04* (3.74–4.36)	2.7* (2.38–3.04)	3.64* (3.42–3.88)
SCs	2.70* (2.57–2.84)	4.13* (3.85–4.43)	3.21* (3.08–3.34)	2.99* (2.84–3.15)	4.03* (3.76–4.31)	3.27* (3.13–3.40)	2.59* (2.41–2.79)	3.25* (2.98–3.55)	2.80* (2.65–2.96)
OBCs	1.92* (1.84–2.00)	2.25* (2.13–2.38)	2.10* (2.04–2.17)	1.97* (1.89–2.06)	2.54* (2.42–2.68)	2.16* (2.09–2.23)	1.64* (1.54–1.75)	2.05* (1.91–2.19)	1.80* (1.72–1.89)
Household Type									
Self employed	1	1	1	1	1	1	1	1	1
Salaried	NA	0.41* (0.39–0.44)	0.31* (0.29–0.32)	NA	0.46* (0.44–0.49)	0.57* (0.55–0.60)	0.31* (0.29–0.34)	0.39* (0.37–0.42)	0.38* (0.36–0.40)
Casual labour	2.38* (2.29–2.46)	2.49* (2.34–2.66)	2.44* (2.37–2.52)	2.29* (2.21–2.37)	2.74* (2.57–2.92)	2.32* (2.25–2.39)	1.92* (1.83–2.02)	2.16* (2.01–2.32)	1.97* (1.89–2.05)
Others	0.55* (0.52–0.58)	0.60* (0.55–0.66)	0.56* (0.53–0.59)	0.51* (0.48–0.54)	0.62* (0.57–0.67)	0.53* (0.51–0.56)	0.83* (0.75–0.92)	0.58* (0.53–0.65)	0.70* (0.65–0.75)

Note: Figures are based on authors' calculations from NSSO data 1999–2000, 2004-05 and 2011–12. * Significant at 1 %; ** Significant at 5 %; NA: Not Available; Figures in parentheses are confidence intervals.

## 5 Results

In a country like India, medical expenditure significantly impacts poverty in the absence of UHC. Standard poverty measures that compare total household expenditure with a stipulated poverty line fail to reflect this fundamental impact adequately. It is unseemly to see that, on one side, there is a significant proportion of the population being classified as non-poor just because of critical OOP health expenditure, which raises their total consumption expenditure above the poverty line (while spending on other non-discretionary items is below the subsistence level). On the other side, high OOP health expenditure is an essential factor that pulls another significant percentage of the poor population into deep poverty.

It is evident from Table 1 that the average household size decreased from 4.9 in 1999-00 to 4.73 in 2004-05 and 4.43 in 2011–12. Rural areas among different sectors, Muslims among the religious groups, OBCs among social groups and self-employed among all household types have reported larger household sizes than their respective counterparts in all three waves of data.

In 1999–2000, 69.9 % of the population reported OOP health expenditure, and this percentage increased to 79.81 % in 2011–12. Among different socioeconomic and demographic categories, rural areas, Muslims, the general category and self-employed reported higher OOP than their respective counterparts in all waves of data.

In 1999–00, health expenditure constituted 4.8 % of the total consumption, which increased to 5.6 % in 2004-05 and 6.8 % in 2011-12 in India. Among different categories, rural areas, Muslims (except 2011–12), SCs (except 2004–05) and others followed by casual labour reported higher health spending as a share of total consumption expenditure than their respective counterparts in all three waves of data. Overall, there is an increase in health expenditure as a share of total consumption expenditure in all categories from 1999-00 to 2011–12, When per capita consumption is already low, and a substantial portion must be allocated to healthcare expenses, it burdens households immensely, increasing their vulnerability to poverty. It is observed from Table 1 that over time, the share of inpatient care in total health expenditure increased. Still, there are a few categories, such as urban areas, other religious categories among different religion types, general categories among all social groups, and salaried households (except 2004–05) among all household types that prefer to get inpatient care as compared to their respective counterparts in all waves of data. Inpatient care is considered the care of the better-off or the last resort of poor households [16]. In all three waves, rural people and casual labourers choose outpatient care compared to their counterparts (See Table 1).

Results show that outpatient care constitutes the most significant share of India's total health expenditure. This burden is driven mainly by households' spending on medicines, which accounted for 61.84 % of 1999-00 and 52.54 % of 2011-12 India's total health expenditure. Also, after medicines, fees and tests, especially in outpatient care, constitute a more significant portion of the country's total health expenditure.

Furthermore, medicines comprise the highest share in inpatient and outpatient care expenditure over all three waves of data. It is worth mentioning that over time, the percentage of medicines on the total spending decreased while the opposite is observed in the fees. The common reasons for this trend could be: 1) the increasing facilities of free medicines in public health facilities, and 2) more dependency on private health care facilities for secondary and tertiary care. Because of the increasing use of private healthcare facilities, the share of fees increased over time.

It is observed from Table 2 that people in rural areas spend more on medicines as compared to their urban counterparts [27,54,55]. The expected reason for this could be that most healthcare facilities are concentrated primarily in urban areas [27,56]. Because of that, people in urban areas get medical consultations at the beginning and prefer prescribed medical treatment. In rural areas, because of the non-availability of healthcare facilities in the vicinity, people choose to get medicines directly from the nearby chemist shops. When the ailment is not treated properly, they go to the hospital for prescribed medicines. It is the main reason for more medical expenditures in rural areas and high spending on consultation fees and tests in the country's urban areas.

Table 3 shows that at all India levels, in 1999–00, 4.6 %, and in 2004–05, 4.5 %, is the percentage of the population above the poverty line (hidden poor) just because of high health expenditure. This percentage constituted 5 % in rural areas in both the data points and 3.4 % and 3.1 %, respectively, in urban areas. In 2011–12, this percentage decreased to 4.1 % at all India levels, contributing 4.7 % in rural and 2.4 % in urban areas. We also found that outpatient care has contributed almost 80 %, 82 % and 76 %, respectively, in this type of poverty, with slightly higher figures in rural areas in all the waves of data. Hidden poverty as a share of pre-poverty increased from 10.5 % in 1999-00 to 12.2 % in 2004-05 and 18.2 % in 2011-12 in India. It is interesting to see here that in rural areas, the share of hidden poor is much more than in the urban areas in all three waves of data. It is clear that the percentage of hidden poor decreased noticeably in urban areas over the period, but this decrease is less visible in rural areas. Hidden poverty as a share of pre-poverty increased substantially in rural, urban and at all India levels. In 1999–00, between rural and urban areas, there was not much difference in the share of outpatient care in hidden poverty, but in 2011–12, this difference widened a lot. In 2011–12, outpatient care contributed 78.7 % in rural and 70.8 % in urban areas to the hidden poverty of the country.

In the same way, removable reinforced poverty as a share of pre-poverty has also increased from 8.2 % in 1999-00 to 12.8 % in 2011-12 at all India levels, with a slightly higher figure for rural areas. The contribution of outpatient care in removable reinforced poverty is more than 85 % in all data waves. Outpatient care contributes more in rural areas than its urban counterparts in removable reinforced poverty figures. This poverty as a share of pre-poverty has increased substantially in rural, urban and at all India levels. In rural areas, these figures have increased from 8.0 % in 1999-00 to 13.1 % in 2011–12, while in urban areas, this increase was from 9.2 % to 11.3 % in the same period.

There is a significant increase in health expenditure-induced poverty (hidden + removable reinforced) in the total poverty from 1999-00 to 2011-12 at rural, urban and all India levels. This figure is more than double in rural areas compared to urban areas in 2011–12. We found a considerable difference in inpatient and outpatient care share in total ill-health-induced poverty. Outpatient care has contributed to an average of four times more than inpatient care in the total poverty impact. This is the total proportion of the population, 97.3 million (as the population of India in 2021 was 1.41 billion^4^), which can be saved from going into poverty if there is UHC in India. Further, the poverty impact of OOP health expenditure has been increasing in proportion to the absolute number of poor. Total health expenditure-induced removable poverty as a share of pre-poverty has risen from 19.0 % in 1999-00 to 30.9 % in 2011–12 (see Table 3).

Table 4 shows the hidden poverty and removable reinforced poverty gap before and after normalising the poverty line in rural, urban and combined areas in India for all data waves. OOP health expenditure has increased hidden poverty gaps from INR 7.3, 4.7 and 6.7 in 1999-00 to 10.3, 6.1 and 9.1 in 2011-12 in rural, urban and country as a whole, respectively. On average, outpatient care contribution is four times higher than inpatient care in this gap in all data waves in the country.

OOP health expenditure has contributed to removable reinforced poverty gap by INR 4.7, 2.9 and 4.2 in 1999-00 and 5.7, 3.6 and 5.1 in 2011-12 in rural, urban and country, respectively. Outpatient care contributed to this gap on an average of 85 % in 1999–20, which increased to 86 % in 2011–12, with a slightly higher figure for rural areas in the country.

Hidden and removable reinforced poverty gap as a share of the pre-poverty gap has also increased over the period in rural, urban and all India levels. It is clear from the figures that, like poverty headcount, the poverty gap has also widened in rural areas compared to the urban counterparts in this period. It is also clear from Table 2 that the total health expenditure-induced poverty gap has also increased from INR 11.0 in 1999-00 to 14.2 in 2011–12, in which outpatient care has contributed more than four times than inpatient care.

To make it more explanatory, we have calculated the normalised poverty gap (NPGI), which shows the gap as a percentage share of the poverty line. The normalised gap has decreased for both hidden poverty and removable reinforced poverty from 1.5 % to 0.9 % and 0.9 to 0.6, respectively, from 1999-00 to 2011-12 for the country as a whole. In the normalised poverty gap, the outpatient contribution is exceptionally higher than inpatient care.

Table 4 also shows the average gap in the form of mean poverty gap impact (MPGI), which means the gap of only those who have reported OOP health expenditure. To make it comparable, we have calculated pre-payment and post-payment MPG and saw the difference. In the case of hidden poverty, this gap has increased from 4.4, 3.4 and 4.3 in 1999-00 to 8.9, 8.2 and 8.7 in 2011-12 for rural, urban and all India levels. Removable reinforced poverty has also increased for urban and all India levels while this figure has come down in the same period for rural areas. In the mean positive gap, outpatient care has contributed the most in all areas and data waves in hidden and reinforced poverty.

The gap as a share of total consumption expenditure has increased in hidden and removable reinforced poverty. In the case of hidden, it has increased from 15.5 % in 1999-00 to 24.3 % in 2011-12 and in the case of removable reinforced, from 9.8 % to 13.7 % between the same period. Table 3, Table 4 show that OOP health expenditure significantly impacts the poverty headcount in all waves of data. Once we realised that health expenditure had affected people above and below the poverty line, the next step was determining which social, religious, and economic categories were affected most by the OOP health expenditure burden.

We did regression analysis and found that Muslims are affected more than other religious groups, including Hindus, in the total poverty impact of OOP health care expenditure. SCs, followed by OBCs among social categories and casual labourers among different household categories, are more vulnerable in rural, urban, and all India levels in the total poverty impact of OOP health expenditure. Over the period from 1999-00 to 2011–12, the burden of ill-health-induced poverty has decreased in the case of Muslims among all religious categories, SCs among all social groups and casual labour among all household types in rural, urban, and all India levels. (see Table 5). Interestingly, the likelihood of casual labour going into poverty due to health expenditure has decreased over time. The odds ratio, which was 2.49, 2.44 and 2.29 in 1999-00 for rural, urban, and all of India, was 1.92, 2.16 and 1.97, respectively, in 2011–12. This change is more pronounced in rural areas than in urban ones. We believe that introducing the Mahatma Gandhi National Rural Employment Guarantee Act (MGNREGA) in 2006 might have contributed to reducing the vulnerability of casual labour, especially in the country's rural areas.

## 6 Discussion

The evidence generated by the study can assist policymakers and healthcare stakeholders in understanding the gravity of the problem and the urgent need for effective interventions. It is evident that OOP health expenditure burdens the population and pushes them into poverty. Outpatient care has contributed the most to the removable poverty impact of health expenditure. This burden is more severe among rural households in the country. This study has also examined the variation in health expenditure-induced impoverishment across different socioeconomic and religious groups. It identified the groups which are more vulnerable to poverty impact. Poverty headcount in rural, urban, and all India has increased significantly when discounting OOP health expenditure from monthly total consumption expenditure in all data waves. It is not only hidden poor people but also a share of reinforced poor, which can be brought out of poverty if there is UHC in the country. A total of 97.3 million people can be carried out of poverty, almost 30 % of India's total poverty. It is worth mentioning that outpatient care has contributed most to hidden and reinforced poverty impact in all waves of data.

The difference in the pre and post-poverty gap has increased substantially from 1999-00 to 2011-12 for rural, urban, and all India levels. When normalised by rural and urban-specific poverty lines, the poverty gap contributed 2.0, 1.0 and 1.5 % due to OOP health expenditure at rural, urban and all India levels, respectively, in 2011–12. In the normalised poverty gap, the share of outpatient care is remarkably high at rural, urban and all India levels.

The mean positive gap also shows a significant increase from 1999-00 to 2011-12 due to OOP health expenditure at rural, urban and all India levels. Socioeconomic and religion-wise explanation of ill-health-induced poverty shows that the population belonging to Muslims among all religions, SCs among all Social categories and Casual labour among all household types have the highest proportion of removable poor.

Four years ago, India launched the most ambitious health mission, the National Health Protection Scheme ‘Ayushman Bharat’, which has two complementary schemes. One provides comprehensive primary care through Health and Wellness Centres. The other aims to protect poor and vulnerable families from financial healthcare in case of inpatient care only [57].

Our results show that it is not inpatient care but outpatient care that contributes most to the impoverishment of households in both rural and urban areas of the country. It contributes heavily to hidden poverty and is the main force pushing a significant percentage of the population into deep poverty in case of reinforced poverty. In a developing country like India, inpatient care is either the care of better-off households or the last resort of poor households. Casual labour and daily wage earners generally avoid inpatient care, leading them to a double burden [58]. In this case, including inpatient care only in the scheme may not protect the poor households from the financial burden and is inadequate to fulfil the country's health needs. The scheme's success needs to be modified appropriately by considering the beneficiaries' social and economic constraints.

Furthermore, in line with other studies such as [59]; our results also revealed that medicines contribute the most to the total financial burden and impoverishment of the households irrespective of the sectors, i.e., rural and urban (see Table 2). There is a need to consider including outpatient care, especially the expenditure on outpatient care medicine, to protect poor households from healthcare's financial burden. This expenditure on medicines accounts for more than 52 % of the country's total health expenditure (see Table 2).

Furthermore, India's disease epidemiology has changed towards the predominance of non-communicable diseases such as diabetes, hypertension, cardiovascular, mental illnesses, and injuries over the past few years [52,59]. Comprehensive primary care delivered in the outpatient setting is necessary for these medical conditions for a long time [58]. It also emphasised the need to include at least a few components of expenditure on outpatient care in NHPS in the country.

There is a great need for improvement in two areas in this insurance-driven financing mechanism for healthcare services. One is the accessibility, cost, and quality check on the services of the private sector [60], along with strengthening the insurance's regulatory framework. Second is the substantial and sustained investment, efficient utilisation of resources, resilient governance, and accountability in the public sector.

As a developing country, India generally has poor health infrastructure and significantly less access to health insurance. Many researchers, such as [61,62]; have emphasised that public health spending has a much more significant effect on health outcomes than economic growth or the people's consumption level. But still, the government's healthcare spending is very low in the country [13]. That is why there has been thoughtful attention from researchers and policymakers to look for different possibilities for expanding health insurance coverage in India.

Though schemes like the National Rural Health Mission (NRHM), the National Urban Health Mission (NUHM), and later the National Health Mission (NHM) were started to provide accessible, affordable and quality healthcare to the population, especially vulnerable groups. They have shown little success in increasing the accessibility of health services, but there is a long way to go to reduce the burden of OOP health expenditure in the country. There is a need to find the reasons for the ineffectiveness of such schemes [63] and improve according to the need to produce desirable results. Quality primary care networking [64] and a system to monitor and evaluate the execution of health insurance schemes [65] are needed from time to time. The single-dimensional approach cannot be the panacea for this problem. Instead, a multidimensional approach can go a long way to protect households from the significant burden of OOP expenditure on critical healthcare in India. There is a need to expand the scope of AB-PMAY to cover outpatient care, especially medicines and more population to an extent, at least until the country has efficiently working health and wellness centres serving every last-mile citizen.

Health insurance security through AB-PMAY can significantly change the poverty level, but focusing only on inpatient care and particular sections of the population may not solve this problem because ill-health-induced poverty is not inpatient care and is group-specific. Socioeconomic and religious group-wise analysis of hidden and reinforced poverty shows that a substantial percentage of the population in different socioeconomic and religious groups have gone into poverty due to the high level of critical OOP health expenditure. In the initial stage, including those who cannot afford it is acceptable. Still, there must be a plan to include every individual under insurance coverage, irrespective of caste and creed. It is essential to make this health financing system more inclusive and sustainable in the long run.

## 7 Conclusion

This study concluded that outpatient care contributes most to the health expenditure impoverishment of households in India, though this type of care has not been included in AB-PMJAY. Muslims, among all religious groups, Scheduled Castes among social groups, and casual labourers among different household groups are more vulnerable to OOP health expenditure-induced removable poverty in the country. Poverty, in general, has dropped significantly, but the share of health expenditure-induced poverty in total poverty has increased substantially. In the past 20 years, it has increased considerably in rural areas and among India's most vulnerable sections. There is a need for universal health insurance coverage, which can make a significant change in the country's poverty rates.

The study revealed that outpatient care contributes significantly to health expenditure-induced poverty in Indian households. However, the government-sponsored health insurance scheme AB-PMJAY has not covered this type of care. This exclusion is making many households vulnerable to the financial consequences of outpatient care. Furthermore, the study revealed that there are specific vulnerable groups, such as Muslims, Scheduled Castes, and casual labourers, who are more susceptible to being pushed into poverty due to out-of-pocket health expenditures in the country. The study observes that while overall poverty in India has decreased, the share of poverty induced by health expenditures has substantially increased. This shift advocates that healthcare costs are becoming a more significant factor in driving poverty levels, particularly in rural areas and among vulnerable sections of the population. We emphasised that universal health insurance coverage is needed in India. Implementing comprehensive health insurance schemes that cover inpatient and outpatient care can help alleviate the financial burden of healthcare expenses on households and reduce poverty rates.

## Limitations of the study

We have analysed the effects of OOP health expenditure on poverty in rural, urban, and all India levels in the form of ill health-induced hidden and reinforced poverty. It is based on the information available in different rounds of consumer expenditure surveys of NSSO. However, these surveys have not been conducted frequently in the country for the last few years. 10.13039/100014337Furthermore, these data sets do not include important information like ailment-wise expenditure, funding sources for health care, different determinants of health, lifestyle choices, and environmental factors, which may provide a more comprehensive understanding of health trends and disparities. Researchers such as [22] have also advocated for improved survey designs for impoverishment work. It will assist researchers to identify trends, changes, and patterns in various factors and enable researchers to access more robust data sets. It will help to track the impact of interventions and policies over time and provide inputs for health financing policy focusing on financial protection for those households immediately above and below the poverty line in the country.

## Funding statement

This research received no grant from any funding agency in the public, commercial or not-for-profit sectors.

## Compliance with ethical standards

The study used a data set that is available online in the public domain; hence, there was no need to seek ethical consent to publish this study.

## Data availability statement

This study is based on anonymized survey data collected by the National Sample Survey Organization (NSSO), a department of the Ministry of Statistics and Programme Implementation, Government of India. Data is available in the public domain and is available in publicly available repositories to individuals both at national and international level through http://www.mospi.gov.in/.

## Additional information

No additional information is available for this paper.

## CRediT authorship contribution statement

**Ramna Thakur:** Writing – original draft, Visualization, Validation, Supervision, Software, Resources, Project administration, Methodology, Investigation, Funding acquisition, Formal analysis, Conceptualization. **Mohammad Ahmad Faizan:** Writing – review & editing, Data curation.

## Declaration of competing interest

The authors declare that they have no known competing financial interests or personal relationships that could have appeared to influence the work reported in this paper.

## Footnotes

1 Etc. is used for further classification within the data sets only.

2 India is a multi-religion country where around 80 % of the population follow Hinduism, 14.2 % adheres to Islam and remaining 6 % follow other religions.

3 The Constitution of India has classified the indigenous population into STs, SCs and OBCs and accorded them special protection. STs and SCs are regarded as socially disadvantaged groups and OBCs are the backward classes.

4 World Bank, (2021). Population-total India. Retrieved from https://data.worldbank.org/indicator/SP.POP.TOTL?locations=IN.
